# Evaluating the efficacy and safety of polyglycolic acid-loading mitomycin nanoparticles in inhibiting the scar proliferation after glaucoma filtering surgery

**DOI:** 10.1080/07853890.2024.2436458

**Published:** 2024-12-05

**Authors:** Tao Li, Juan Tang, Changfen Li, Guogang Liu, Ying Li, Shanlan Guo, Qilin Fang, Jing Li, Xing Qi, Xingde Liu, Juan Du, Dan Zhang, Silun Xiong, Jiaqian Li, Yueyue Tan, Biao Li, Chuanqiang Dai, Qinqin Zhang, Jiaman Li, Xiaoli Wu

**Affiliations:** a Department of Ophthalmology, Zi Yang Central Hospital, Sichuan, China; b Department of Ophthalmology, Key Laboratory of Ophthalmology, Zi Yang Central Hospital, Sichuan, China; c Department of Endocrinology, Zi Yang Central Hospital, Sichuan, China; d Department of Pathology, Zi Yang Central Hospital, Sichuan, China; e Department of Dermatology, Zi Yang Central Hospital, Sichuan, China; f Department of Experimental Medicine, Zi Yang Central Hospital, Sichuan, China; g Department of Medical Education, Zi Yang Central Hospital, Sichuan, China; h Department of Anesthesia Operation Center, Zi Yang Central Hospital, Sichuan, China

**Keywords:** Mitomycin, PLGA, glaucoma filtering surgery, scar proliferation, inflammation

## Abstract

**Purpose:**

To prepare a polyglycolic acid-loaded mitomycin drug (MMC-ATS-@PLGA) to inhibit scar proliferation after glaucoma filtering surgery (GFS) *via* an anti-inflammatory mechanism that minimally affected intraocular pressure, which provided another therapeutic strategy for this disease.

**Methods:**

We first detected the physicochemical properties of MMC-ATS-@PLGA. Next, we tested the biosafety of MMC-ATS-@PLGA *in vivo* and *in vitro*. Then, we assessed the therapeutic effects of MMC-ATS-@PLGA by laboratory and clinical examinations.

**Results:**

In this study, we synthesized a new type of nanomedicine (MMC-ATS-@PLGA) with good stability and biocompatibility for inhibiting scar proliferation after GFS. The break-up time (BUT), Schimer test and intraocular pressure changes in GFS rabbits before and after treatment with MMC-ATS-@PLGA were not significantly different. Three weeks after GFS, the MMC-ATS-@PLGA group displayed significant decreases in nuclear volume, corneal cell oedema, type I and III collagen fibre expression, normal organelle morphology and collagen fibre arrangement. Compared with those in the FML and MMC groups, the α-SMA, CTGF and type III collagen fibres in the MMC-ATS-@PLGA group decreased more significantly, indicating that MMC-ATS-@PLGA can effectively inhibit the expression of these inflammatory factors during the inhibition of scar proliferation after GFS.

**Conclusion:**

We successfully synthesized MMC-ATS-@PLGA, which could effectively inhibit scar proliferation after GFS *via* anti-inflammatory effects but had little effect on intraocular pressure. This new type of nanomedicine has good biosafety and stability and is worthy of further exploration in clinical practice.

## 1. Introduction

Glaucoma is an eye disease characterized by optic nerve atrophy and visual field defects [1,2]. This disease is the world’s first irreversible blinding eye disease and is usually accompanied by high intraocular pressure [3]. The number of glaucoma patients worldwide is expected to increase to 118 million by 2040 [4]. Currently, the main surgical treatment for glaucoma is filtering surgery, but scar formation in the filtering passage is the main reason for surgical failure [5,6]. Some research has shown that the expression of inflammatory factors such as connective tissue growth factor (CTGF), type III collagen fibres and α-smooth muscle actin (α-SMA) is increased after glaucoma filtering surgery, thus inducing the proliferation and transformation of fibroblasts into myofibroblasts in conjunctival and scleral tissues, which is a key cause of scar formation after glaucoma filtration surgery [7–9]. Therefore, for patients undergoing glaucoma filtration surgery, timely and regular treatment of scar formation in the filtration channel is crucial [10]. Currently, corticosteroids are the main method for preventing and treating scar formation in the filtration channel after glaucoma surgery [11]. However, the long-term use of corticosteroids can lead to complications such as high intraocular pressure and cataracts, which limits their clinical use [12]. Therefore, it is important to develop a new drug aimed at blocking scar tissue proliferation after glaucoma filtration surgery that has minimal side effects [13].

Mitomycin C (MMC) has a significant antifibrotic effect and has been used in glaucoma filtration surgery for more than 30 years [14,15]. The use of MMC greatly improves the success rate of glaucoma filtration surgery, but its clinical application is limited due to serious complications such as wound leakage, low intraocular pressure, corneal toxicity, and intraocular infection, which can pose a new threat to the already damaged visual function in patients [16,17]. At the same time, due to the natural physiological and physical barriers of the human eye, drugs have a short residence time on the ocular surface and a low drug utilization rate [18,19]. Therefore, further exploring how to reduce the toxic side effects of MMC, prolong the drug’s residence time on the ocular surface, and more effectively inhibit scar proliferation after glaucoma filtration surgery is worthwhile [20,21].

Following the application of nanotechnology in the field of ophthalmology, its advantages, such as easy modification, sustained release, targeting and good biocompatibility, have been applied to solve related clinical problems [22,23]. Polylactic acid hydroxyacetic acid copolymer (PLGA) is a biodegradable functional polymeric organic compound composed of two monomers, namely, lactic acid and hydroxyacetic acid, that has good biocompatibility, no toxicity, good performance in capsule and film formation [24,25]. This nanomaterial is widely used in pharmaceuticals, medical engineering materials, and modern industrial fields [26]. Therefore, this study aimed to prepare a new nano eye drop with low toxicity and high anti-inflammatory effects by attaching MMC to PLGA, and further explored the ability of the new nanodrug to inhibit scar proliferation after glaucoma filtration surgery through *in vivo* and *in vitro* experiments.

## 2. Methods

### 2.1. Synthesis of MMC-ATS-@PLGA

MMC-ATS-@PLGA was prepared using a modified double-emulsion method (W/O/W method). According to this method, 50 mg of polylactic/PLGA nano­particles and 10 mg of MMC (mitomycin C) were fully dissolved in 50 mL of methanol solution. Then, 200 μL of double-distilled water was added to serve as the inner aqueous phase, and the mixture was emulsified with a sonicator (Sonics & Materials Inc., Newtown, Connecticut, USA) to obtain a primary emulsion. After ultrasonic uniform dispersion in an ice bath, the liquid was subjected to cryogenic centrifugation at 12,000 rpm for 10 min. The supernatants were removed, and the sediments were collected and resuspended in PBS or ATSs. Finally, MMC-ATS-@PLGA was purified by centrifugation (10,000 rpm, 5 min).

### 2.2. Characterization of MMC-ATS-@PLGA

The nanostructure of MMC-ATS-@PLGA was observed by transmission electron microscopy (TEM; Hitachi H-7600, Hitachi, Ltd, Tokyo, Japan). The average size, polydispersity index (PDI), and surface potential were measured by a dynamic light scattering detector (DLS, Malvern Instruments, Malvern, UK). To test the stability of VHPK-PLGA@COL, the size and PDI were monitored using DLS in phosphate-buffered saline (PBS) for prolonged durations (1, 2, 3, 4, 5, 6, and 7 days). The absorbance of the resulting methanol solutions containing different concentrations of mitomycin was measured using a UV–vis-NIR spectrophotometer (UV2600, Shimadzu, Japan), the absorption peak was determined based on the maximum absorbance value, and the corresponding concentration absorbance standard curves were calculated. The drug loading capacity (LC) and encapsulation efficiency (EE) were calculated as follows:

$$\begin{array}{c} \begin{array}{c} \text{LC(\%) = mass of MMC encapsulated on nanoparticles/}\\ \text{mass of nanoparticles }\times \text{ 100\%} \end{array}\\ \begin{array}{c} \text{EE(\%) = mass of MMC encapsulated on nanoparticles/}\\ \text{mass of MMC used }\times \text{ 100\%} \end{array} \end{array}$$

The mass of MMC encapsulated in nanoparticles = mass of MMC used-mass of MMC in the supernatant.

### 2.3. The establishment of an inflammatory cell and animal model after glaucoma filtration surgery

Human conjunctival fibroblasts (HCFs) were purchased from Procell (Wuhan, China), cultured in DMEM supplemented with 10% FBS and 1% penicillin/streptomycin and incubated at 37 °C in a 5% CO_2_ atmosphere. TGF-β_1_ (5 ng/mL) was used to induce inflammation to establish the cell model of GFS at 37 °C for 24 h.

Forty six New Zealand white rabbits (purchased and raised from the Animal Research Center of Chongqing Medical University) weighing 2.0–2.5 kg were selected, and all rabbits were quarantined and acclimatized for one week before the experiments. All rabbits underwent glaucoma filtering surgery. The animal handling process strictly followed the requirements of the Institutional Animal Care and Use Committee (IACUC) of Chongqing Medical University. All animal experiments were approved by the Animal Ethics Committee of Chongqing Medical University. Then, all rabbits were randomly divided into the control group, FML group, MMC group, and MMC-ATS-@PLGA group. The remaining rabbits not subjected to glaucoma filtering surgery were regarded as the normal group. For all animals, the right eye was chosen as the experimental eye. The concentration of FML was 0.1 mg/mL, while the concentrations of MMC in the MMC group and the MMC-ATS@PLGA group were the same (0.1 mg/mL).

### 2.4. The *in vitro* cytotoxicity of MMC-ATS-@PLGA

The toxicity of FML, MMC or MMC-ATS-@PLGA was detected by CCK-8 assays and live/dead cell staining. First, HCFs were seeded in a 96-well plate at a density of 1 × 10^5^ cells per well and incubated for 24 h. The cells were treated with FML, MMC or MMC-ATS-@PLGA at different concentrations (2, 4, 6, 8, 10, 20, 40 and 80 μg/mL) for 24 h. The cells that were not treated were regarded as the control group. The CCK-8 assay was used to test cell viability. In addition, HCFs (5 × 10^5^) were seeded in laser confocal cell culture dishes and cultured for 24 h, followed by treatment with 6 µg/mL FML, MMC or MMC-ATS-@PLGA for 24 h.

### 2.5. Biosafety testing of MMC-ATS-@PLGA on a rabbit eye surface

The rabbits were randomly and investigator-blindly divided into 4 groups (G1–G4: normal, FML, MMC, and MMC-ATS-@PLGA; 5 mice/group). These groups were treated with 30 μL of ATS suspension, FML-ATS, MMC-ATS, or MMC-ATS-@PLGA suspension topically three times a day. The concentrations of MMC administered to every rabbit in the MMC-ATS group and the MMC-ATS-@PLGA group were the same (0.1 mg/mL). After three weeks of treatment, the rabbits were anesthetized *via* an auricular vein injection of pentobarbital sodium (3%, 1 mL/kg). Then, 2 μL of 1% fluorescein sodium was used to observe corneal epithelial staining and tear film rupture time under a slit lamp. In addition, Schirmer test paper (TQM Schirmer, Indian) was placed into the conjunctival sac at the external third of the lower eyelid for 5 min without topical anaesthesia, and the tear secretion on the ocular surface of the rabbits was recorded. Moreover, a handheld rebound tonometer (Tono-Pen AVIA, Reichert, CA, USA) was used to measure the intraocular pressure of rabbits under the influence of FML, MMC, and MMC-ATS-@PLGA. Then, the cornea was fixed with paraformaldehyde (4% in PBS) for 1 h, embedded in paraffin and cut into sections. Thin tissue slices (8 mm thick) were stained with H&E to observe the macroscopic and microstructural changes in the cornea under the influence of FML, MMC, and MMC-ATS-@PLGA.

### 2.6. Evaluation of the anti-inflammatory effect by flow cytometry

HCFs were seeded on six-well plates at a density of 5.0 × 10^5^ cells/mL and cultured in DMEM supplemented with 10% FBS and 1% penicillin/streptomycin containing TGF-β_1_ (5 ng/ml) to induce cellular inflammatory transformation. After 24 h of incubation, the cells were divided into FML, MMC, and MMC-ATS-@PLGA groups. The concentrations of MMC used in the MMC group and the MMC-ATS-@PLGA group were the same (6 μg/mL). After 24 h of drug treatment, the HCFs in each group were detached from the six-well plates, washed with PBS, resuspended in 500 μL of binding buffer, and incubated with FITCconjugated annexin V (Sanjian, Hangzhou, China) and propidium iodide (Sigma) at room temperature in the dark for 15 min. Then, the cell apoptosis rate in each group was detected by flow cytometry.

### 2.7. Immunofluorescence

After establishing the inflammatory cell model, the inflammatory HCFs were divided into the control group, FML group, MMC group and MMC-ATS-@PLGA group. The concentrations of FML, MMC and MMC-ATS-@PLGA were the same (6 μg/mL). Cell immunofluorescence was performed according to the following process: experimental group: cell slide→cell culture and fixation→cell membrane rupture→sealing off (PBS:goat serum = 1:10)→primary antibody incubation (PBS:antibody = 2:100)→secondary antibody incubation (PBS:antibody = 2:100)→DAPI staining→slicing microscope observation. Finally, fluorescence images were obtained *via* immunofluorescence microscopy (Leica SP8, Germany). Then, ImageJ software was used to calculate the fluorescence signal intensity of the inflammatory cytokines α-SMA and CTGF and type III collagen fibres in the cytoplasm.

### 2.8. Analysis of the therapeutic effect on tissue morphology

After three weeks of treatment with different reagents, the formation of filter bubbles in each group of rabbits after glaucoma filtration surgery was observed and recorded under a slit lamp microscope. Then, all rabbits were sacrificed, and conjunctival and scleral tissues from the control (ATS), FML, MMC and MMC-ATS-@PLGA groups were extracted for histological and immunohistochemical analysis. The cellular microstructural changes in the conjunctival and scleral tissues were observed and recorded under a light microscope (DMi1, Leica, Solms, Germany).

### 2.9. Evaluation of anti-inflammatory effects by enzyme-linked immunosorbent assay (ELISA) and RT–qPCR

According to the experimental design, the conjunctival and scleral tissues of rabbits were extracted three weeks after treatment with FML, MMC or MMC-ATS-@PLGA. Then, proteins and RNA were extracted from conjunctiva and scleral tissues for subsequent experimental use. Finally, the expression levels of α-SMA, CTGF and type III collagen fibres in the conjunctival tissue were measured by commercial enzyme-linked immunosorbent assay (ELISA) kits according to the manufacturer’s instructions, and the expression levels of these inflammatory cytokines in the scleral tissue were tested by reverse transcription–PCR (RT–PCR).

### 2.10. Statistical analysis

The data are presented as the means ± SDs. Statistical analysis was performed with GraphPad Prism version 8.3.0 (GraphPad Software, CA, USA) through multiple t tests, row means with SDs, simple linear regression, ordinary oneway ANOVA and two-way ANOVA. Statistical significance was set to *p* < 0.05.

## 3. Results

### 3.1. Observation of the physicochemical properties of MMC-ATS-@PLGA

**Figure 1.**
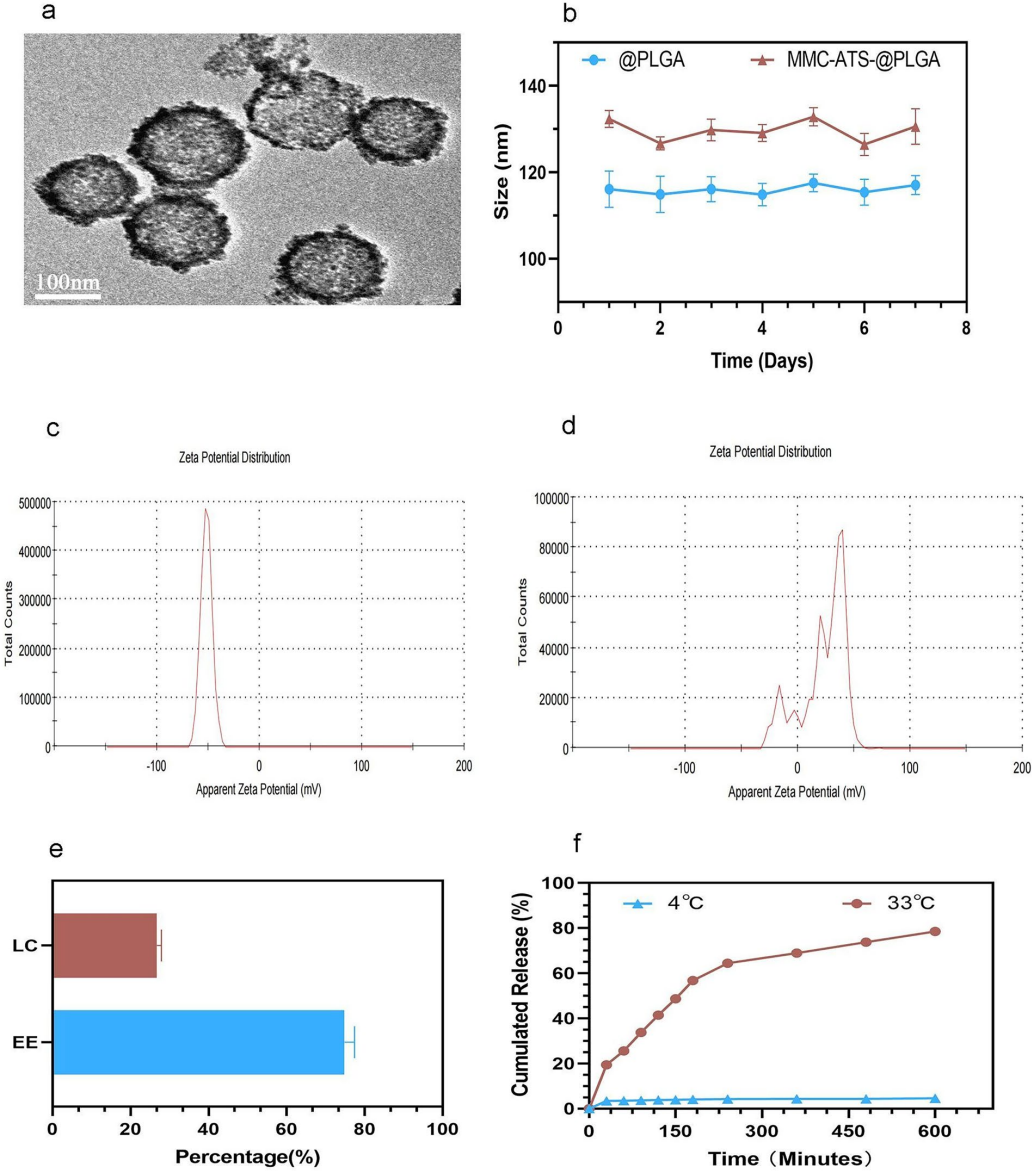
(a) TEM image of MMC-ATS-@PLGA (200 μg/mL). (b) 1–7 day particle size monitoring chart of @PLGA and MMC-ATS-@PLGA (200 μg/mL). (c) Zeta potential distribution of @PLGA (200 μg/mL). (d) Zeta potential distribution of MMC-ATS-@PLGA (200 μg/mL). (e) the LC and EE of MMC-ATS-@PLGA. (f) *In vitro* cumulative release of MMC from MMC-ATS-@PLGA in ATS solution at 4 °C and 33 °C. *T* tests and row means with SDs were performed for analysis (*p <* 0.001).

As shown in Figure 1a, MMC-ATS-@PLGA had a relatively regular spherical core shell structure, and MMC adhered to the surface of the nanospheres. In addition, our research indicated that the 0.1% MMC solution was transparent and was resuspended in ATS, while MMC-ATS-@PLGA appeared milky white at the same concentration of MMC. Moreover, changes in the sizes of the @PLGA and MMC-ATS-@PLGA in PBS were detected for seven days, and the results showed that the physical properties of MMC-ATS-@PLGA was stable (Figure 1b). Moreover, the zeta potentials of @PLGA and MMC-ATS-@PLGA were −67.28 ± 6.84 mV and 36.49 ± 4.25 mV, respectively (Figure 1c,d). The results indicated that the surface of MMC-ATS-@PLGA had a positive charge after drug modification, which could better adapt to the negatively charged environment of the ocular surface. Moreover, the standard UV absorption spectrum curves showed that MMC and MMC-ATS-@PLGA exhibited a characteristic absorption peak at 282 nm, while @PLGA exhibited no such characteristic absorption peak. Then, we calculated that the MMC encapsulation efficiency (EE) and loading capacity (LC) were approximately 72.48% and 31.65%, respectively, according to the standard curve of the MMC absorption spectra (Figure 1e). Furthermore, the cumulative release of MMC-ATS-@PLGA was calculated according to the calibration curve of MMC generated by HLPC (Figure 1f), and the results showed that the cumulative release rates of MMC were 4.76 ± 0.31% and 78.38 ± 2.42% after 600 min at 4 °C (storage temperature) and 33 °C (ocular surface temperature), respectively. These results showed that MMC could be easily released from MMC-ATS-@PLGA at corneal temperature, but it was difficult for MMC to be released at storage temperature.

### 3.2. Viability of HCFs treated with MMC-ATS-@PLGA Formulations

**Figure 2.**
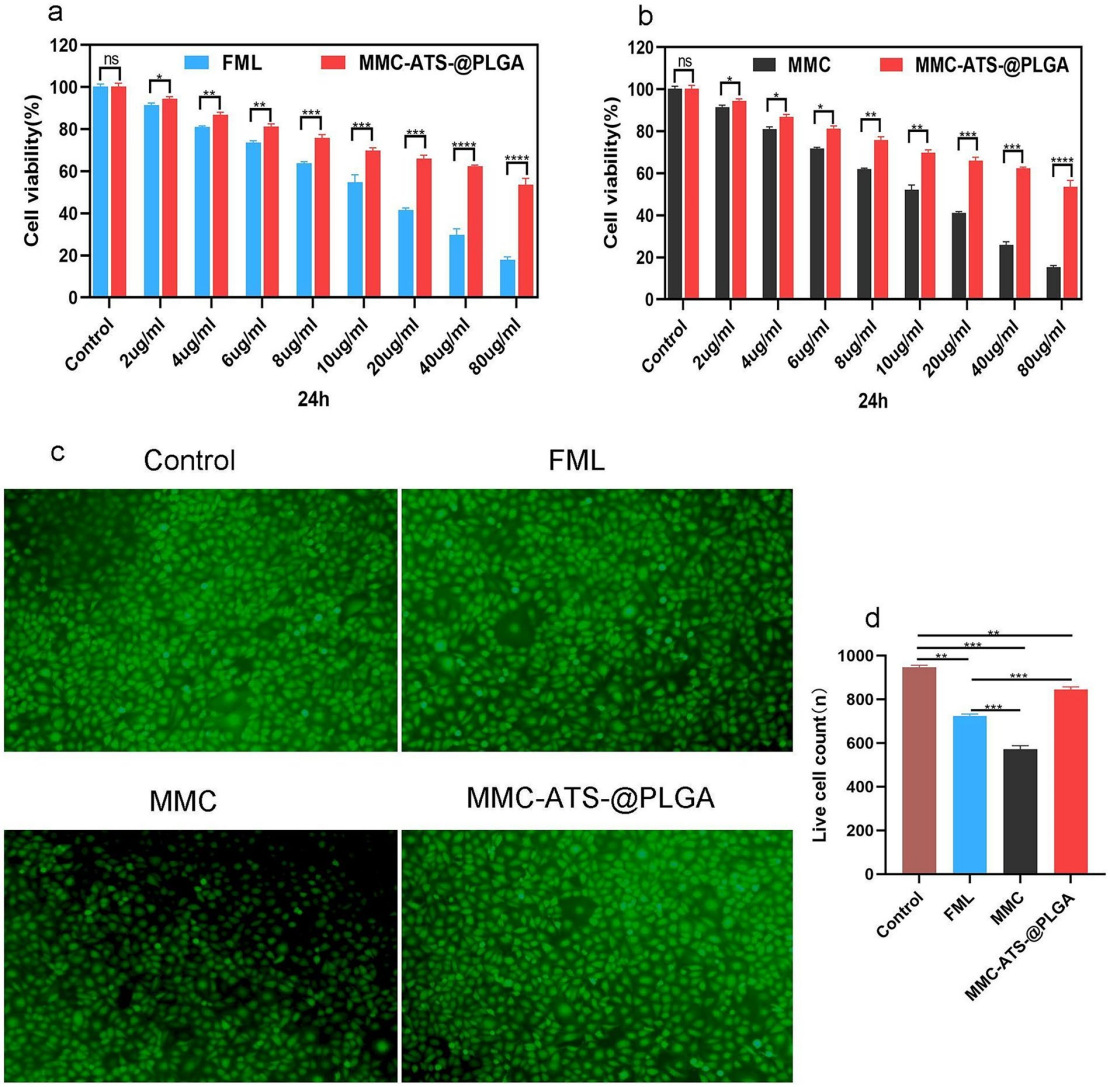
(a, b) Viability of HCFs after treatment with various concentrations of FML, MMC and MMC-ATS-@PLGA for 24 h. (c, d) Live/dead staining images of HCFs treated for 24 h with MMC, FML and MMC-ATS-@PLGA (6 µg/mL). Ordinary one-way ANOVA was performed (*p < 0.05, **p < 0.01, ***p < 0.001, ***p < 0.0001).

As shown in Figure 2, the cytotoxicity of free MMC and MMC-ATS-@PLGA was evaluated by assessing the viability of HCFs using a CCK-8 assay and cell viability staining. Our research showed that cell viability gradually decreased as the concentrations of FML and MMC increased. However, the viability of the cells in the MMC-ATS-@PLGA group decreased slowly. At the maximum drug concentration of 80 μg/mL, the percentages of viable cells in the FML group, MMC group and MMC-ATS-@PLGA group were 18.47 ± 1.65%, 16.72 ± 1.43% and 58.83 ± 2.75%, respectively (Figure 2a,b). The concentration of MMC was fixed at 6 µg/mL, and in live/dead staining images, the prevalence of green fluorescent cells (live cells) in the MMC-ATS-@PLGA group suggested that this nanomedicine was safer than FML and MMC for HCF cells (Figure 2c,d). These results indicated that the good biological effects of nanotechnology can effectively reduce the cytotoxicity of MMC.

### 3.3. Biological Safety of the rabbit eye surface after treatment with MMC-ATS-@PLGA Formulations

**Figure 3.**
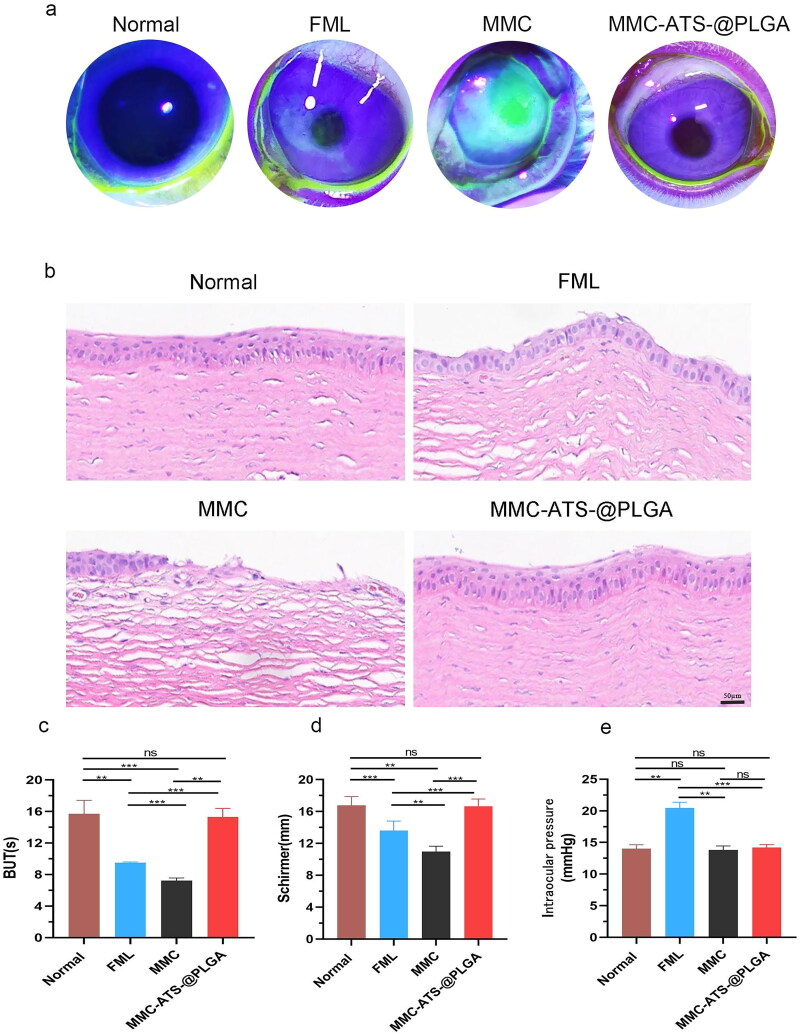
(a) Corneal staining images of the normal, FML, MMC, and MMC-ATS-@PLGA groups after one week of drug treatment. (b) Morphological changes in the corneal epithelial layer in the normal, FML, MMC, and MMC-ATS-@PLGA groups after one week of drug treatment. (c). The results of tear film rupture time (but) in the normal, FML, MMC, and MMC-ATS-@PLGA groups after one week of drug treatment. (d) The results of the tear secretion test (Schirmer) in the normal, FML, MMC, and MMC-ATS-@PLGA groups after one week of drug treatment. (e) Differences in intraocular pressure in the normal, FML, MMC, and MMC-ATS-@PLGA groups after three weeks of drug treatment. Ordinary one-way ANOVA was performed (*n* = 5, *p < 0.05, **p < 0.01, ***p < 0.001, ****p < 0.0001).

As shown in the corneal staining images (Figure 3a), compared with those in the normal group, the corneas in the FML, MMC, and MMC-ATS-@PLGA groups exhibited different manifestations after 3 weeks of treatment. The MMC group showed diffuse punctate staining due to the toxic side effects of the drugs on the corneal epithelium. However, the toxic side effects of the MMC drug combined with nanocarriers were significantly reduced, and no significant corneal fluorescence was observed in the MMC-ATS-@PLGA group because of the good biocompatibility of the PLGA nanomaterial. After 3 weeks of drug treatment, corneal tissue was further extracted for HE staining. As shown in Figure 3b, compared with that in the normal group, the corneal epithelial layer in the MMC group was significantly thinner, with a decrease in the number of epithelial cells, irregular cell morphology, and significant tissue oedema. Nevertheless, the MMC-ATS-@PLGA group did not exhibit any significant changes, as mentioned above, and its morphology was similar to that of the normal group. Furthermore, we analyzed differences in intraocular pressure and surface tear severity among rabbits after three weeks of drug treatment. The average tear film rupture time (BUT) in the normal group was 15.48 ± 0.79 s, while that in the FML (9.36 ± 0.82 s) and MMC (7.83 ± 0.57 s) groups both showed a downward trend, but there was no statistically significant difference between the MMC-ATS-@PLGA group (15.16 ± 0.54 s) (*p* < 0.05) (Figure 3c). At the same time, the tear secretion time (Schirmer) showed the same trend as the BUT (Figure 3d). Further analysis of the impact of FML, MMC and MMC-ATS-@PLGA on intraocular pressure revealed that, compared with those in the normal group (14.27 ± 0.79 mmHg), there was no significant difference in intraocular ­pressure between the MMC (14.12 ± 0.84 mmHg) and MMC-ATS-@PLGA groups (14.46 ± 0.85 mmHg), while there was a significant increase in intraocular pressure in the FML group (20.72 ± 1.43 mmHg) (*p* < 0.05) (Figure 3e). These results suggested that long-term use of MMC-ATS-@PLGA not only had no toxic side effects on the corneal surface but also had no significant impact on intraocular pressure.

### 3.4. Evaluation of the anti-inflammatory effect at the cell level

**Figure 4.**
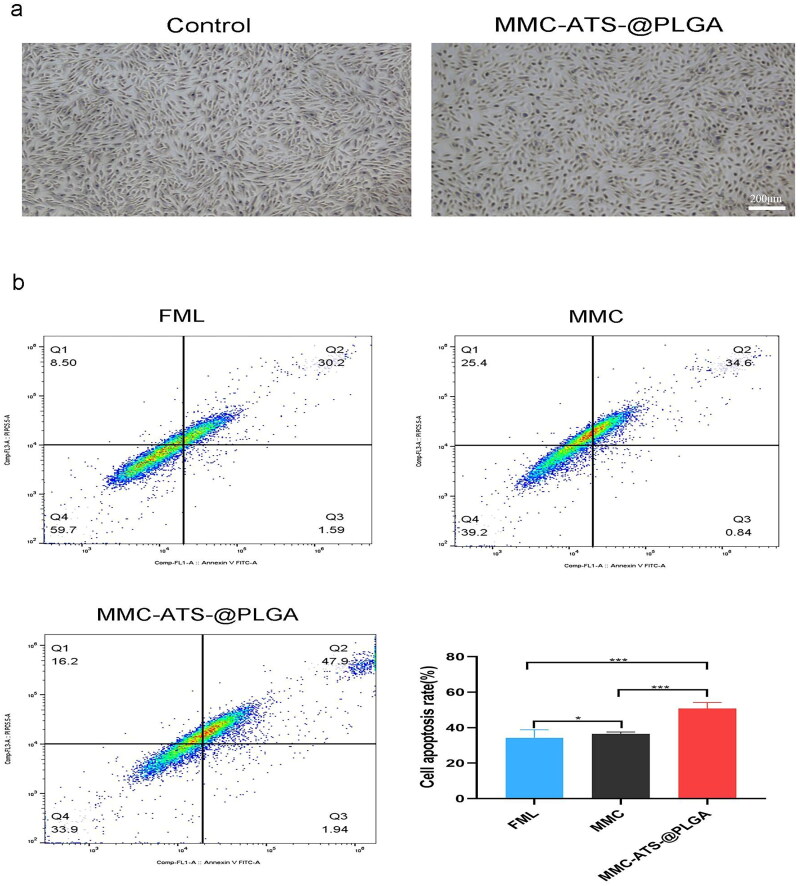
(a) Changes in the quantity and morphology of inflamed HCFs after incubation with MMC-ATS-@PLGA for 24 h (MMC: 6 µg/mL). (b) The apoptotic rate of inflammatory HCFs was measured in the MMC, FML and MMC-ATS-@PLGA groups after incubation for 24 h (6 µg/mL) (*n* = 3; *p < 0.05, **p < 0.01, ***p < 0.001, ****p < 0.0001).

The inflammatory-inducible factor TGF-β_1_ was used to induce the transformation of HCFs into inflammatory cells to establish a GFS model *in vitro*. As shown in Figure 4a, after 24 h of drug treatment, the number of inflamed HCFs in the MMC-ATS-@PLGA group significantly decreased, the cell morphology decreased, and nuclear pyknosis occurred compared with those in the control group. Further analysis of the apoptotic rate of inflammatory HCFs after 24 h of drug treatment *via* flow cytometry revealed that compared with that in the FML group (31.47 ± 1.89%), the apoptotic rate of inflammatory HCFs in the MMC group (35.73 ± 1.46%) and MMC-ATS-@PLGA group (49.64 ± 2.83%) increased to varying degrees, while that in the MMC-ATS-@PLGA group was the highest apoptotic rate of inflammatory HCFs (Figure 4b).

### 3.5. Anti-inflammatory effect in cellular immunofluorescence

**Figure 5.**
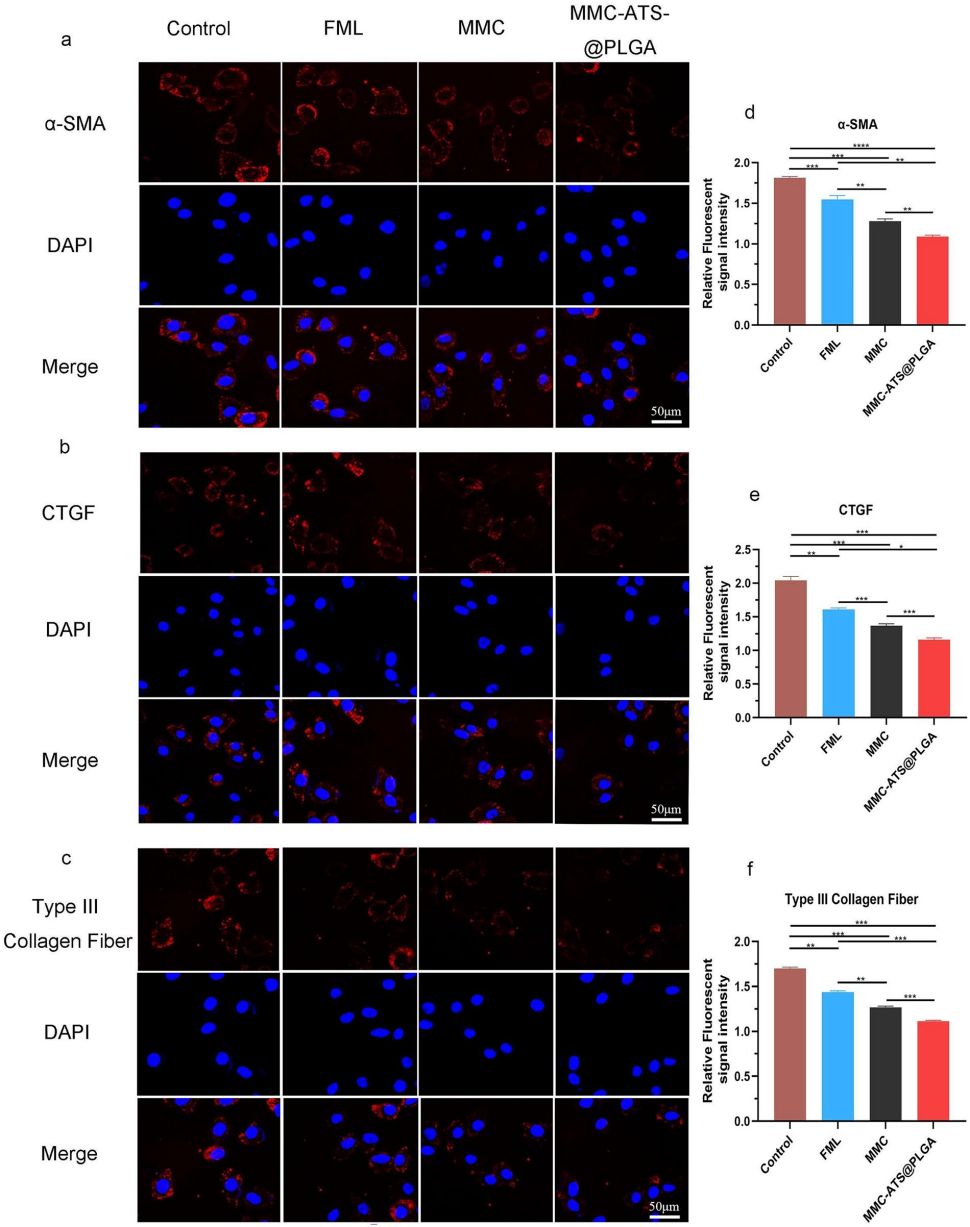
(a–c) Images of immunofluorescence staining for the α-SMA, CTGF and Type III collagen fibre antibody in the MMC, FML and MMC-ATS-@PLGA groups after incubation for 24 h. (d–g) Statistical analysis of the immunofluorescence signals of the α-SMA, CTGF and Type III collagen fibre antibody in the MMC, FML and MMC-ATS-@PLGA groups after incubation for 24 h (6 µg/mL) (*n* = 3; *p < 0.05, **p < 0.01, ***p < 0.001, ****p < 0.0001).

As shown in Figure 5a–c, the signal intensity of cell fluorescence in the control group was greater than that in the other groups regardless of the presence of the α-SMA, CTGF and Type III collagen fibre antibodies. After treatment with FML, MMC or MMC-ATS-@PLGA, the expression of these inflammatory factors decreased, and the most significant decrease occurred in the MMC-ATS-@PLGA group. Furthermore, ImageJ software was used to analyze the cellular immunofluorescence images, and the intensities of these antibodies in the control, FML, MMC and MMC-ATS-@PLGA groups were as follows: (1) α-SMA (Figure 5d): 1.82 ± 0.17, 1.54 ± 0.13, 1.34 ± 0.18, 1.12 ± 0.09; (2) CTGF (Figure 5e): 2.07 ± 0.15, 1.62 ± 0.18, 1.41 ± 0.13, 1.20 ± 0.14; and (3) Type III Collagen Fibre (Figure 5f): 1.71 ± 0.08, 1.46 ± 0.12, 1.28 ± 0.11, 1.13 ± 0.07. The above results indicated that FML, MMC and MMC-ATS-@PLGA can all effectively inhibit the expression of the aforementioned inflammatory factors, and the MMC-ATS-@PLGA treatment had the greatest inhibitory effect.

### 3.6. Morphological analysis of the therapeutic effects of GFS *in vivo*

**Figure 6.**
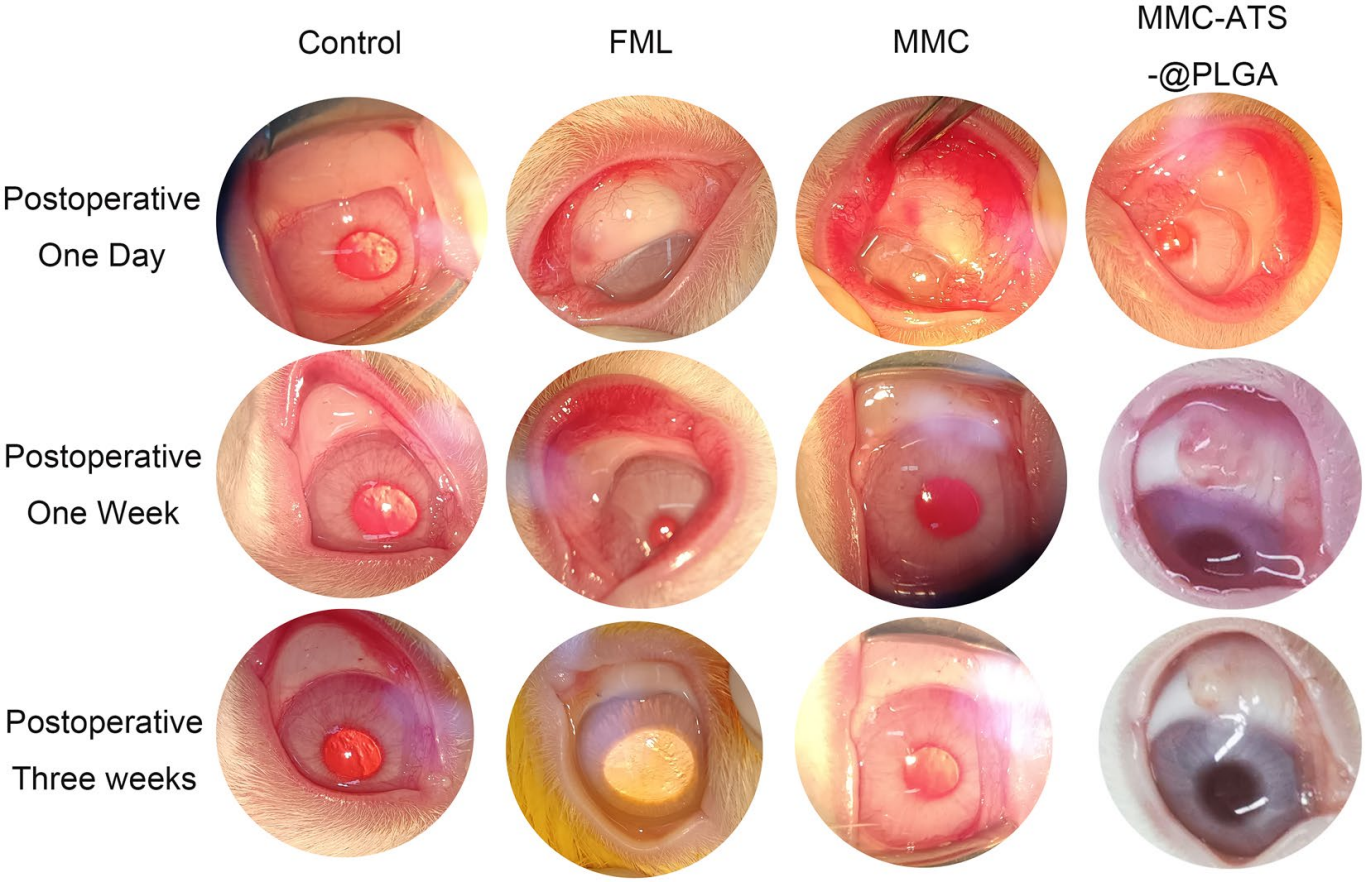
Changes in functional filtration bubbles after GFS surgery at different times after treatment with FML, MMC or MMC-ATS-@PLGA.

On the first day after GFS surgery, there were no complications, such as corneal defects, anterior chamber exudation, or filtration bubble leakage, in any group. Functional filtration bubbles were formed in all groups, and there was no significant difference in morphology. As shown in Figure 6, compared with those in the first week after surgery, the filtering bubbles in the control group, FML group, MMC group, and MMC-ATS-@PLGA group decreased by 12.78 ± 2.93 mm^2^, 10.43 ± 2.58 mm^2^, 8.57 ± 1.64 mm^2^, and 7.03 ± 1.45 mm^2^, respectively. The FML, MMC and MMC-ATS-@PLGA groups exhibited elevated and dispersed follicles and a significant reduction in congestion. As time progressed, the filtering bubbles gradually decreased and flattened. Compared with that two weeks after surgery, the filtering bubbles in the control group, FML group, MMC group, and MMC-ATS-@PLGA group decreased by 21.49 ± 2.37 mm^2^, 14.85 ± 2.27 mm^2^, 12.38 ± 1.95 mm2, and 9.46 ± 1.73 mm^2^, respectively, at three weeks after surgery. The area of the filtering bubbles in the MMC-ATS-@PLGA group was significantly larger than that in the FML group and MMC group, and the filtering bubbles in the control group basically disappeared.

**Figure 7.**
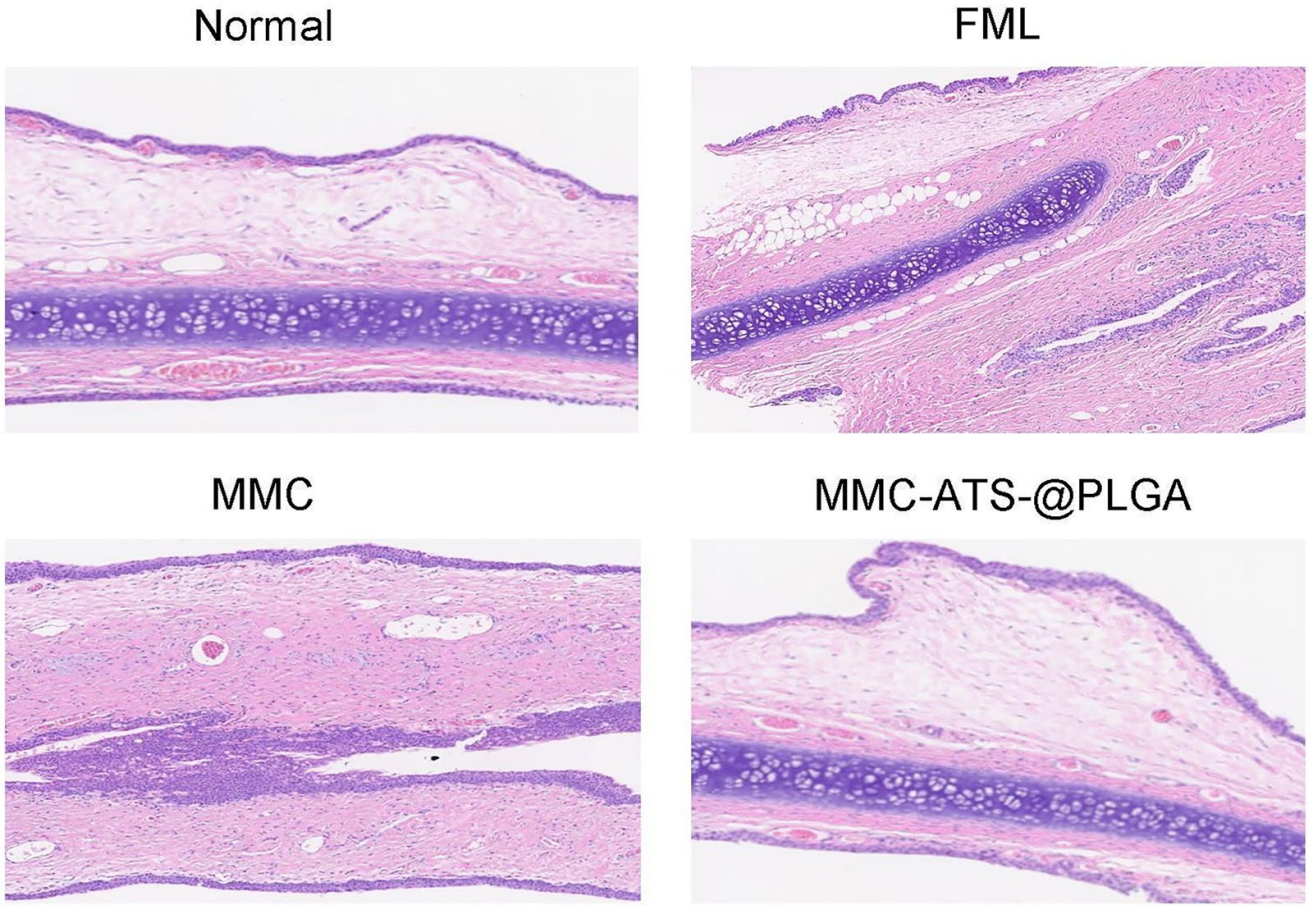
Changes in the conjunctiva after GFS surgery at different times after treatment with FML, MMC or MMC-ATS-@PLGA.

**Figure 8.**
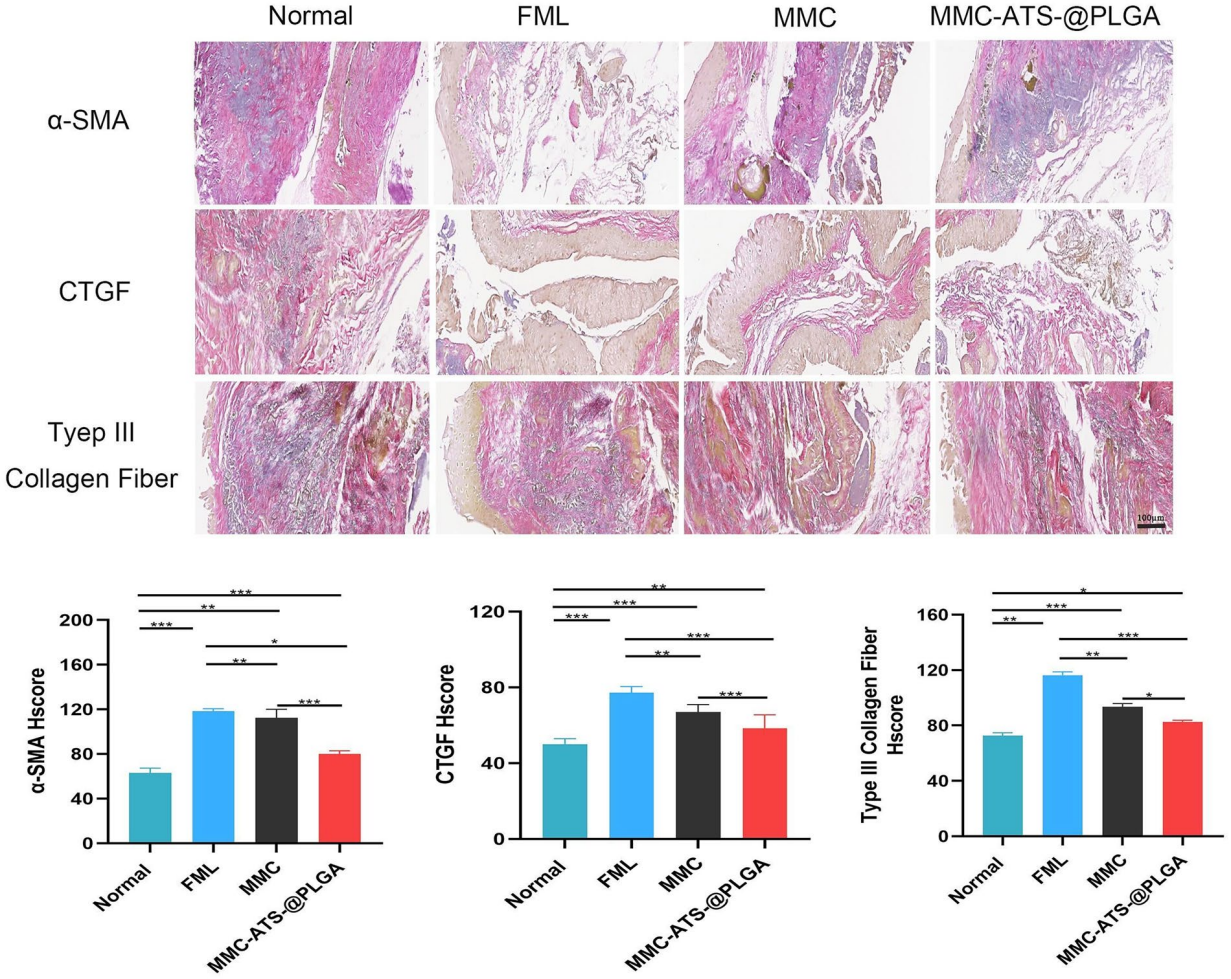
Changes in the scleral flap after GFS surgery at different times after treatment with FML, MMC and MMC-ATS-@PLGA according to immunohistochemistry.

**Figure 9.**
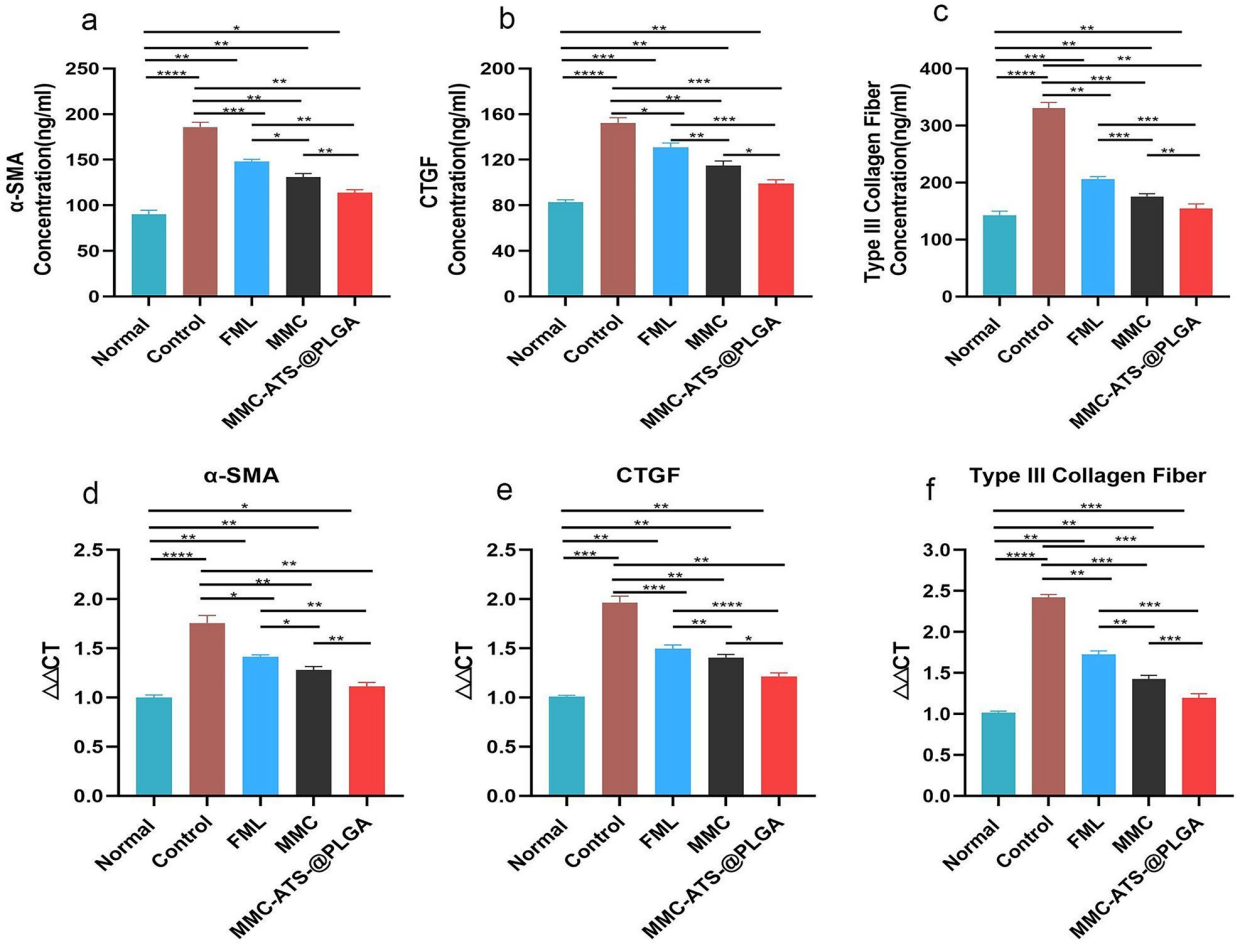
(a–c) The expression of α-SMA, CTGF and type III collagen fibres antibodies in conjunctival tissue in the normal, control, FML, MMC and MMC-ATS-@PLGA groups after 3 weeks of treatment. (d–f) Gene translation of the α-SMA, CTGF and type III collagen fibres in scleral tissue in the normal, control, FML, MMC and MMC-ATS-@PLGA groups after 3 weeks of treatment in the model rabbits at the gene level. Ordinary one-way ANOVA was performed (*p < 0.05, **p < 0.01, ***p < 0.001, ****p < 0.0001).

Three weeks after GFS surgery, the conjunctiva and scleral flap in the filtering bleb area of the rabbit eye were further extracted for pathological tissue analysis. The results showed that in both the scleral and conjunctival tissues, a large amount of generated collagen fibres was observed in the control group, and their arrangement was irregular. The collagen fibres were dense and thick, and their arrangement was disordered in the control group. However, the FML group, MMC group, and MMC-ATS-@PLGA group had fewer collagen fibres, with relatively regular morphology and neat arrangement; in particular, in the MMC-ATS-@PLGA group, the morphology of the conjunctiva and sclera tissues was similar to that of the normal group (Figures 7 and 8). Then, the expression of α-SMA, CTGF and type III collagen fibres was measured directly in all groups by immunohistochemistry in the 3rd week after GFS surgery. Furthermore, we statistically analyzed the H-scores of the inflammation-related antibodies as follows: H-score=∑(pi × i)=(percentage with weak intensity × 1)+(percentage with moderate intensity × 2)+(percentage with strong intensity × 3). For instance, compared with the H-scores of α-SMA expression in the FML (118.37 ± 2.58) and MMC (112.86 ± 2.83) groups, the H-score in the MMC-ATS@PLGA (79.51 ± 2.39) group showed the most obvious decrease (Figure 9), which was close to that of the normal group (63.67 ± 1.92). Surprisingly, the expression H-scores of type III collagen fibres and CTGF showed the same trend.

### 3.7. Evaluation of the anti-inflammatory effect of animal conditions *via* proteomics and genomics

Furthermore, conjunctival tissue proteins were extracted from the animal models for ELISA, and the results suggested that compared with those in the normal group, the expression levels of α-SMA, CTGF and Type III collagen fibre antibodies in the control group were significantly higher (Figure 9a–c). After treatment with FML, MMC or MMC-ATS-@PLGA for three weeks, the expression of inflammatory factors gradually decreased, and that in the MMC-ATS-@PLGA group significantly decreased (*p* < 0.05). Furthermore, the translation of the α-SMA, CTGF and Type III collagen fibre genes in GFS rabbit sclera was analyzed by PCR (Figure 9d–f), and the results showed that all these genes were downregulated after treatment with FML, MMC and MMC-ATS-@PLGA; in particular, in the MMC-ATS-@PLGA group, the downregulation of these genes was most significant (*p* < 0.05).

## 4. Discussion

Glaucoma is one of the most common blinding eye diseases [27]. The elevated intraocular pressure of patients causes a series of visual impairments, such as visual field defects, optic disc depression, and ultimately complete blindness [28,29]. Currently, glaucoma filtering surgery is the most effective treatment method for mid- to late-stage glaucoma, but postoperative scarring of filtering follicles is the main reason for the failure of this surgery [30,31]. Currently, it is widely believed that scar formation is mainly related to increased collagen fibre synthesis and the proliferation of fibroblasts and myofibroblasts [32]. Currently, there are many drugs used for preventing scar formation after glaucoma surgery, and these drugs can be divided into the following categories: glucocorticoids, anti-metabolic drugs, collagen crosslinking inhibitors, anticoagulants, and anti-vascular endothelial growth factor drugs [33,34]. The most common drugs among them are antimetabolic drugs. For example, mitomycin, the most commonly used antimetabolic drug, inhibits fibre proliferation and prevents the formation of filtering blister scars [35]. However, its clinical application is limited due to serious complications such as wound leakage, low intraocular pressure, corneal toxicity, and intraocular infection [36].

Polylactic acid hydroxyacetic acid copolymer (PLGA) has long been studied, and its advantages of reducing toxicity and prolonging the action time of the encapsulated drug have been fully exploited as a carrier for drugs that treat anterior eye disease [37–39]. Therefore, we hypothesized that MMC can effectively inhibit the expression of inflammatory factors related to GFS. Furthermore, we used PLGA as a nanodrug carrier with the advantages of easy modification, sustained release, targeting, and good biocompatibility to develop a new drug aimed at preventing scar formation after glaucoma filtration surgery with fewer side effects [40].

In this study, we first detected the physicochemical properties of MMC-ATS-@PLGA immediately after its preparation. The particle size, morphology, zeta potential, and UV spectrum results of MMC-ATS-@PLGA all showed that MMC was successfully loaded onto PLGA. The increase in the mean size over time confirmed the stability of MMC-ATS-@PLGA, and the results showed that it was stable when stored at 4 °C and exhibited good release performance at 33 °C. In addition, the encapsulation efficiency and loading capacity of MMC-ATS-@PLGA indicated the successful preparation of a sustainable release system.

To verify the *in vitro* safety of nanomedicine after the combination of MMC and PLGA nanocarriers, CCK-8 and cell live/dead staining were employed to compare the toxicity of FML, MMC and MMC-ATS-@PLGA on HCFs. As the drug concentration increased, the viability of the cells treated with FML and MMC gradually decreased, but the viability of the cells treated with MMC-ATS-@PLGA remained relatively stable. Then, we used 6 µg/mL FML, MMC and MMC-ATS-@PLGA to perform live/dead cell staining, and the prevalence of green fluorescent cells indicated that MMC-ATS-@PLGA was significantly safer than FML and MMC. Subsequently, we further analyzed the safety of MMC-ATS-@PLGA from an *in vivo* experimental perspective. After 3 weeks of drug treatment on the surface of the rabbit eye, diffuse patchy staining of the corneas in the MMC group, slight patchy staining of the corneas in the FML group, and no obvious fluorescence staining of the basic corneal epithelium were observed in the corneas in the MMC-ATS-@PLGA group. After further extraction of corneal tissue for HE staining, we discovered various degrees of thinning of the corneal epithelium in the FML and MMC groups, with the most severe thinning in the MMC group. However, there was no significant change in the morphology of the corneal epithelium in the MMC-ATS-@PLGA group. To analyze the effect of drugs on tear secretion on the ocular surface, the BUT and Schirmer tests were used in this study, and the results showed that there was no difference in the BUT or Schirmer test between the MMC-ATS-@PLGA group and the normal group, while both the FML and MMC groups had smaller tear secretion than the MMC-ATS-@PLGA group. Moreover, compared with the long-term use of FML, which can easily cause an increase in intraocular pressure, this study indicated that MMC-ATS-@PLGA had no effect on intraocular pressure. In summary, from both *in vivo* and *in vitro* experimental perspectives, the excellent biosafety of nanotechnology can effectively reduce the drug toxicity and side effects of MMC.

Then, we assessed the anti-inflammatory effects of the MMC-ATS-@PLGA formulations *in vitro*. First, the inflammatory inducible factor TGF-β_1_ was used to induce the transformation of HCFs into inflammatory cells to establish a GFS model. After coincubation with drugs and inflamed HCFs for 24 h, the number of cells in the MMC-ATS-@PLGA group significantly decreased, the cell morphology became smaller, and nuclear pyknosis occurrence increased compared with those in the control group. Moreover, the flow cytometry results further indicated that MMC-ATS-@PLGA can effectively promote the apoptosis of inflamed HCFs in the GFS cell model, and its effect was superior to that of FML and MMC. Research has shown that surgical trauma stimulates increased expression of TGF-β_1,_ which can further promote the generation of α-SMA, CTGF and Type III collagen fibres [41,42]. In addition, α-SMA is an important component of myofibroblasts, and its high expression in myofibroblasts can activate the process of scar formation after glaucoma filtering surgery [43]. To our delight, as we can see from the cellular immunofluorescence results, the expression of inflammatory factors, including α-SMA, CTGF and Type III Collagen Fibre, was downregulated in the inflamed HCFs after treatment with MMC-ATS-@PLGA. The above results indicated that nanomedicines can effectively inhibit the proliferation of inflamed HCFs.

Further analyzing the trend of changes in filtering blebs in different drug groups after glaucoma filtration surgery at 1–3 weeks, we discovered that the filtration blebs in the control group basically disappeared, while those in the FML and MMC groups were small and flat. However, the filtration blebs in the MMC-ATS-@PLGA group bulged, and their size met the experimental requirements. Further analysis of changes in conjunctival and scleral tissues in the surgical area revealed that compared to the disordered arrangement of collagen fibres in the control group, the morphology of conjunctival and scleral tissues in the MMC-ATS-@PLGA group was similar to that in the normal group. In addition, ELISA of conjunctival tissue demonstrated that MMC-ATS-@PLGA can effectively reduce the expression of inflammatory factors, including α-SMA, CTGF and Type III collagen fibre. Moreover, the PCR results showed the same trend.

## 5. Conclusion

In summary, this study utilized the excellent biological effects of a PLGA nanocarrier to successfully reduce the toxicity of MMC and synthesized a new type of nanomedicine (MMC-ATS-@PLGA). This nanomedicine that can effectively promote the apoptosis of inflammatory HCFs, thus inhibiting the expression of α-SMA, CTGF and Type III Collagen Fibre, thereby effectively preventing postoperative scar formation in the filter passage after glaucoma filtration surgery. In addition, the MMC-ATS-@PLGA drug had little effect on surface tears and intraocular pressure. Therefore, the development of nanodrugs for preventing scar formation after glaucoma filtration surgery is worthy of further exploration.

## Supplementary Material

Supplemental Material .pdf

## Data Availability

Data will be made available on request.

## Arrive guideline statement

This study adhered to the ARRIVE guidelines (https://arriveguidelines.org/).
